# Supplementary material for: PCB defect detection based on pseudo-inverse transformation and YOLOv5
Source: PLoS One. 2024 Dec 12;19(12):e0315424. doi: 10.1371/journal.pone.0315424 (PMC11637289; doi:10.1371/journal.pone.0315424)
Supplement: S1 File — (DOC) [file pone.0315424.s001.doc]

**Table 1 Figure 6(a) Original data set**

| Defects | Instances |
| --- | --- |
| Missing hole | 2202 |
| Mouse bite | 2222 |
| Open circuit | 2110 |
| Short | 2102 |
| Spur | 2122 |
| Sporious copper | 2132 |

**Table 2 Figure 8 Original data set**

| Image | Fuzzy kernel =1 | | | | Fuzzy kernel =2 | | | |
| --- | --- | --- | --- | --- | --- | --- | --- | --- |
|  | TwIST-1 | TwIST-2 | SB-1 | SB-2 | TwIST-1 | TwIST-2 | SB-1 | SB-2 |
| 1 | 37.43 | 37.72 | 38.48 | 38.30 | 36.89 | 37.31 | 37.57 | 37.6 |
| 2 | 39.33 | 39.92 | 39.64 | 39.99 | 37.25 | 38.21 | 39.21 | 39.2 |
| 3 | 40.63 | 40.62 | 40.58 | 40.98 | 39.87 | 39.97 | 40.13 | 40.13 |
| 4 | 39.21 | 40.13 | 39.86 | 40.13 | 38.70 | 39.14 | 39.51 | 39.50 |
| Image | Fuzzy kernel =4 | | | | Fuzzy kernel =6 | | | |
|  | TwIST-1 | TwIST-2 | SB-1 | SB-2 | TwIST-1 | TwIST-2 | SB-1 | SB-2 |
| 1 | 37.23 | 37.53 | 37.92 | 37.85 | 37.62 | 37.75 | 38.32 | 38.45 |
| 2 | 39.00 | 39.31 | 39.50 | 39.50 | 38.92 | 39.14 | 39.79 | 39.8 |
| 3 | 40.26 | 40.30 | 40.36 | 40.38 | 40.49 | 40.48 | 40.62 | 40.62 |
| 4 | 39.98 | 39.52 | 40.51 | 40.52 | 39.19 | 39.57 | 39.82 | 39.82 |

**Table 3 Figure 9 Original data set**

| Image | Fuzzy kernel =1 | | | | Fuzzy kernel =2 | | | |
| --- | --- | --- | --- | --- | --- | --- | --- | --- |
|  | TwIST-1 | TwIST-2 | SB-1 | SB-2 | TwIST-1 | TwIST-2 | SB-1 | SB-2 |
| 1 | 64.87 | 65.24 | 29.98 | 29.54 | 44.98 | 55.00 | 25.47 | 25.78 |
| 2 | 49.56 | 35.45 | 25.76 | 26.01 | 15.03 | 32.89 | 22.40 | 22.40 |
| 3 | 54.21 | 32.78 | 26.54 | 25.37 | 49.88 | 36.76 | 22.43 | 22.43 |
| 4 | 29.30 | 41.35 | 27.47 | 24.10 | 28.97 | 37.13 | 23.35 | 23.35 |
| Image | Fuzzy kernel =4 | | | | Fuzzy kernel =6 | | | |
|  | TwIST-1 | TwIST-2 | SB-1 | SB-2 | TwIST-1 | TwIST-2 | SB-1 | SB-2 |
| 1 | 55.80 | 59.87 | 27.58 | 27.58 | 61.58 | 55.56 | 27.53 | 27.53 |
| 2 | 32.22 | 44.68 | 25.42 | 25.43 | 44.56 | 32.17 | 25.34 | 25.3 |
| 3 | 51.22 | 47.36 | 26.78 | 26.77 | 48.76 | 40.24 | 25.78 | 25.75 |
| 4 | 30.00 | 41.65 | 25.43 | 25.45 | 30.26 | 44.65 | 25.22 | 25.22 |

**Table 4 Figure 10 Original data set**

| - | TwIST-1 | TwIST-2 | SB-1 | SB-2 |
| --- | --- | --- | --- | --- |
| 1. PSNR | | | | |
| 0 | 22 | 22 | 22 | 22 |
| 10 | 34 | 34 | 37 | 34 |
| 20 | 37 | 38 | 41 | 38 |
| 30 | 40 | - |  | - |
| 40 | 40 | - |  | - |
| 50 | 40 | - |  | - |
| 60 | 40 | - |  | - |
| 1. the iteration step size | | | | |
| Step | 59 | 39 | 37 | 37 |

**Table 5 Figure 12(a) Original data set**

| Value (%) | 0 | 40 | 80 | 120 | 160 | 200 |
| --- | --- | --- | --- | --- | --- | --- |
| Precision | 95.00 | 98.25 | 98.27 | 98.37 | 98.37 | 98.37 |
| Recall | 95.00 | 98.65 | 99.16 | 99.24 | 99.24 | 99.24 |
| mAP/IoU=0.5 | 95.00 | 98.75 | 98.85 | 99.15 | 99.15 | 99.15 |

**Table 6 Figure 13 Original data set (%)**

| Defects | SSD | YOLOv3 | YOLOv4 | YOLOv5 | Faster R-CNN | T-YOLOv5 |
| --- | --- | --- | --- | --- | --- | --- |
| Missing hole | 71.42 | 82.51 | 96.42 | 97.84 | 95.47 | 99.41 |
| Mouse bite | 72.38 | 78.89 | 87.54 | 94.46 | 91.61 | 98.81 |
| Open circuit | 72.53 | 89.24 | 97.51 | 99.41 | 96.42 | 99.55 |
| Short | 71.57 | 77.16 | 88.39 | 95.69 | 98.63 | 9.52 |
| Spur | 79.14 | 88.43 | 94.78 | 95.82 | 92.16 | 99.56 |
| Sporious copper | 73.16 | 79.97 | 89.22 | 94.58 | 92.41 | 98.89 |
