# Supplementary material for: PCB defect detection based on pseudo-inverse transformation and YOLOv5
Source: PLoS One. 2024 Dec 12;19(12):e0315424. doi: 10.1371/journal.pone.0315424 (PMC11637289; doi:10.1371/journal.pone.0315424)
Supplement: Attachment — Submitted filename: Response to reviewers comments.doc [file pone.0315424.s002.doc]

Reviewers' comments:

Reviewer's Responses to Questions

Comments to the Author

1. Is the manuscript technically sound, and do the data support the conclusions?

The manuscript must describe a technically sound piece of scientific research with data that supports the conclusions. Experiments must have been conducted rigorously, with appropriate controls, replication, and sample sizes. The conclusions must be drawn appropriately based on the data presented.

Reviewer #1: Partly

Reviewer #2: Partly

Reply: Thank you very much for your comments. This paper begins with a detailed review of existing PCB defect detection techniques and YOLOv5 applications, highlighting their strengths and weaknesses. The proposed methodology is then presented, with innovations and improvements clearly explained. The experimental setup and evaluation metrics are thoroughly described to ensure that the reader can fully understand and replicate the study. Experimental results are provided in sufficient detail, including tables and graphs that help visualise the results.

2. Has the statistical analysis been performed appropriately and rigorously?

Reviewer #1: No

Reviewer #2: Yes

Reply: Thank you very much for your comments. The statistical analysis of the experimental results have been supplemented in the manuscript. As follows:

Finally, the DNN-based PCB-DD method proposed in reference [4] and the CNN-based PCB-DD method proposed in reference [6] are presented and compared with T-YOLOv5. Based on these literatures, the study proposes optimal parameters. The performance is compared and the test is repeated five times to get the t-test results as shown in Table 5.

**Table 5. Performance comparison of different methods**

| Model | Recall | | Testing time | | Accuracy | |
| --- | --- | --- | --- | --- | --- | --- |
|  | *t*-value | *p*-value | *t*-value | *p*-value | *t*-value | *p*-value |
| T-YOLOv5 vs DNN | 2.35 | <0.001 | 3.45 | <0.001 | 2.41 | <0.001 |
| T-YOLOv5 vs CNN | 4.56 | <0.001 | 4.87 | <0.001 | 3.59 | <0.001 |

In Table 5, the *t*-values between the proposed method and other methods are all above 1.96 (at 95% confidence level, two-tailed test). It shows that the differences between different methods are statistically significant. Concurrently, the results illustrate the efficacy and superiority of the proposed method.

3. Have the authors made all data underlying the findings in their manuscript fully available?

The PLOS Data policy requires authors to make all data underlying the findings described in their manuscript fully available without restriction, with rare exception (please refer to the Data Availability Statement in the manuscript PDF file). The data should be provided as part of the manuscript or its supporting information, or deposited to a public repository. For example, in addition to summary statistics, the data points behind means, medians and variance measures should be available. If there are restrictions on publicly sharing data—e.g. participant privacy or use of data from a third party—those must be specified.

Reviewer #1: No

Reviewer #2: Yes

Reply: Thank you very much for your comments. All data underlying the findings described in this manuscript have been provided.

4. Is the manuscript presented in an intelligible fashion and written in standard English?

PLOS ONE does not copyedit accepted manuscripts, so the language in submitted articles must be clear, correct, and unambiguous. Any typographical or grammatical errors should be corrected at revision, so please note any specific errors here.

Reviewer #1: Yes

Reviewer #2: No

Reply: Thank you very much for your comments. The language of the manuscript has been improved by some scholars who have published English papers.

5. Review Comments to the Author

Please use the space provided to explain your answers to the questions above. You may also include additional comments for the author, including concerns about dual publication, research ethics, or publication ethics. (Please upload your review as an attachment if it exceeds 20,000 characters)

Reviewer #1: Comments on manuscript ID PONE-D-24-40368:

Title: “PCB Defect Detection Based on Pseudo-inverse Transformation and YOLOv5”

Some key suggestions are listed below:

1.There are certain grammatical and misspelling issues noted throughout the text, like “TOLOv5 is further improved using Transformer”. Please check and make sure to address them.

Reply: Thank you very much for your comments. The grammar and spelling mistakes have been checked and proofread. Please see some examples as follows:

Secondly, Transformer is introduced to improve YOLOv5, and the batch normalization and network loss function are optimized.

2. The abstract is not well written. The abstract could benefit from a more concise presentation of key points. Some sentences feel lengthy and complex, which can reduce readability.

Reply: Thank you very much for your comments. The abstract has been rewritten, and the revised abstract mainly included research background, the proposed method, experimental results, conclusions and contributions. As follows:

With the development of integrated circuit packaging technology, the layout of printed circuit boards has become complicated. Moreover, the traditional defect detection methods have been difficult to meet the requirements of high precision. Therefore, in order to solve the problem of low efficiency in defect detection of printed circuit boards, a defect detection method based on pseudo-inverse transform and improved YOLOv5 is proposed. Firstly, a defect image restoration model is constructed to improve image clarity. Secondly, Transformer is introduced to improve YOLOv5, and the batch normalization and network loss function are optimized. These methods improve the speed and accuracy of PCB defect detection. Experimental verification showed that the restoration speed of the image restoration model was 37.60%-42.38% higher than other methods. Compared with other models, the proposed PCB defect detection model had an average increase of 10.90% in recall and 12.87% in average detection accuracy. The average detection accuracy of six types of defects in the self-made PCB data set was over 98.52%, and the average detection accuracy was as high as 99.1%. The results demonstrate that the proposed method can enhance the quality of image processing and optimize YOLOv5 to improve the accuracy of detecting defects in printed circuit boards. This method is demonstrably more effective than existing technology, offering significant value and potential for application in industrial contexts. Its promotion could facilitate the advancement of industrial automation manufacturing.

3. The key contributions of your proposed method are missing in introduction section.

Reply: Thank you very much for your comments. The contribution of the proposed method has been explained in introduction section. Please see the details as follows:

An IR model based on PIT is developed to improve the clarity of PCB defect images. By combining total variation (TV) and shear transformation (ST), this model effectively removes image noise and preserves important details. At the same time, Transformer is introduced to improve YOLOv5, which enhances the ability to extract model features, thus improving the ability to identify PCB defects. By optimizing the batch normalization (BN) and loss function in YOLOv5, the DA of the model is improved. The PCB-DD method proposed in this paper has important application value and significance in the actual PCB-DD field, which is conducive to improving the efficiency and accuracy of PCB-DD in the manufacturing industry. The research not only advances the theoretical development of PCB defect detection technology but also demonstrates clear practical advantages, offering an efficient and precise solution for the detection of defects in industrial automation manufacturing.

4. The methodology should explain how the PCB-DD dataset was constructed, including details about the number of samples, types of defects included, resolution of images, and data augmentation techniques. This information is critical for replicability and for understanding the dataset’s role in enhancing model performance. It would be useful to compare the proposed PCB-DD dataset against existing publicly available datasets (if any exist) to demonstrate the model's applicability in broader scenarios.

Reply: Thank you very much for your comments. Details such as sample number, defect type, image resolution and data expansion technology of PCB data are shown in Section 2.3. In addition, the data type distribution and object center point distribution of the proposed PCB data set have been supplemented in the manuscript, as follows:

The PCB-DD dataset contains 1386 images, covering 6 different types of defects, including pin holes, mouse bites, open circuits, short circuits, dispersion, and miscellaneous copper. These images can be used for image detection, classification, and registration tasks. The images in the dataset are captured by industrial cameras with a resolution of 1920×1080, and the dataset is divided into training, validation, and testing sets in a ratio of 6:2:2. The format of the dataset follows the PASCAL VOC format.

The visual analysis of PCB data set is shown in Fig. 6.

**Figure 6. Analysis chart of pcb number series**

Figures 6(a) and 6(b) show the sample distribution of the PCB dataset and the position of the center of the object. There are 11,601 images in the training set and 1,289 images in the test set. There are 11,601 images in the training set and 1,289 images in the test set.

And in order to further illustrate the applicability of the model in a wider range of scenarios, the study introduces the publicly available PCB-DD (D1) and the self-constructed PCB-DD (D2) from Peking University for the comparison of the model training effect. The details are shown in Table 2.

**Tabel 2. Performance comparison of different dataset models**

| Data set | Accuracy (%) | Precision (%) | Recall (%) | F1 Score (%) |
| --- | --- | --- | --- | --- |
| D1 | 94.57 | 94.53 | 92.33 | 93.42 |
| D2 | 99.20 | 98.97 | 97.87 | 98.42 |

As can be seen from Table 2, the proposed model has superior training results in the self-built dataset D2, while the model accuracy and F1 score in the unimproved public dataset D1 are above 93%. This indicates that the model is better adapted to the specific features of PCB defect detection and has strong generalisation ability.

5. The conclusion can benefit from a broader reflection on the potential impact of the proposed model beyond the experiment. How might this technology affect industries that rely on PCB production? What future applications, besides just PCB-DD, could benefit from the advancements in the model?

Reply: Thank you very much for your comments. The influence of the proposed method on PCB industry and other applications of the model have been supplemented in the conclusion of the manuscript. As follows:

By more accurately detecting defects in PCBs, it is possible to reduce the number of defective products reaching the market, thereby improving the reliability and quality of the final product. Automated and efficient defect detection can reduce the cost of manual inspection and potentially reduce material waste and rework due to defects. As electronic devices become more complex, PCBs need to be designed and manufactured with greater precision. This technology can support more complex and detailed PCB designs. In addition, fast and accurate defect inspection can shorten the time it takes to get a product from design to market.

In addition, the T-YOLOv5 model is being considered for application in areas such as security monitoring and medical image analysis to improve the accuracy of lesion detection and public safety. In precision agriculture, the model can be used to monitor crop health, detect pests and diseases, and automate harvesting. In environmental monitoring, the model can be used to detect and classify different types of pollution or signs before natural disasters.

Reviewer #2: Title: “PCB Defect Detection Based on Pseudo-inverse Transformation and YOLOv5”

Some key suggestions are:

1. Some misspellings and grammatical issues noted throughout the text. Please check and make sure to address them all.

Reply: Thank you very much for your comments. The misspellings and grammatical issues have been solved. The whole manuscript has been checked and the laguage has been improved.

2. The term "pseudo-inverse transformation" is used without prior context. Since it is a key component of the method, a brief explanation or rationale for choosing this approach would enhance understanding for readers unfamiliar with this technique.

Reply: Thank you very much for your comments. The pseudo-inverse transformation has been explained as follows:

Moreover, PIT is introduced to improve the TV fidelity term to obtain the expression formula of the PCB-IR model. In mathematics, if a matrix with the same number of rows and columns is reversible, it has an inverse matrix. Therefore, the result of multiplying the matrix with its inverse matrix is the identity matrix. However, for non-square matrices or irreversible square matrices (such as singular matrices), the inverse cannot be obtained directly. At present, PIT can be used to solve this problem. PIT is not only applicable to irreversible matrices, but also to invertible matrices.

3. The mention of "Transformer is introduced to improve YOLOv5" is quite brief. It would be helpful to provide a bit more insight into how the Transformer architecture is incorporated and why it enhances the performance of YOLOv5 in this specific context.

Reply: Thank you very much for your comments. Transformer's self-attention mechanism can operate on the whole image and capture the global context information. This is particularly beneficial for elucidating the interrelationships and interactions between disparate objects within a PCB image, thereby facilitating more accurate identification of the relationships between different defects. In addition, multiple downsampling in YOLOv5 can lead to the loss of important information in the high-level feature map. Transformer can supplement this information, and through its encoder-decoder architecture, it can reintegrate the low-level detailed information with the high-level semantic information.

4. The abstract can be reorganized slightly to follow a more typical structure: problem statement, method, results, and significance.

Reply: Thank you very much for your comments. The abstract has been rewritten, and the revised abstract mainly included research background, the proposed method, experimental results, conclusions and contributions. As follows:

With the development of integrated circuit packaging technology, the layout of printed circuit boards has become complicated. Moreover, the traditional defect detection methods have been difficult to meet the requirements of high precision. Therefore, in order to solve the problem of low efficiency in defect detection of printed circuit boards, a defect detection method based on pseudo-inverse transform and improved YOLOv5 is proposed. Firstly, a defect image restoration model is constructed to improve image clarity. Secondly, Transformer is introduced to improve YOLOv5, and the batch normalization and network loss function are optimized. These methods improve the speed and accuracy of PCB defect detection. Experimental verification showed that the restoration speed of the image restoration model was 37.60%-42.38% higher than other methods. Compared with other models, the proposed PCB defect detection model had an average increase of 10.90% in recall and 12.87% in average detection accuracy. The average detection accuracy of six types of defects in the self-made PCB data set was over 98.52%, and the average detection accuracy was as high as 99.1%. The results demonstrate that the proposed method can enhance the quality of image processing and optimize YOLOv5 to improve the accuracy of detecting defects in printed circuit boards. This method is demonstrably more effective than existing technology, offering significant value and potential for application in industrial contexts. Its promotion could facilitate the advancement of industrial automation manufacturing.

5. The methodology does not explain how PIT is applied or why it was chosen over other traditional methods like Fourier transforms or wavelet-based methods. Including a brief explanation of why PIT is particularly well-suited for PCB image restoration would strengthen the methodology.

Reply: Thank you very much for your comments. The choose and the application of PIT have been explained as follows:

Although Fourier transform and wavelet based methods are very effective in image processing, they usually need to make a global assumption about the image degradation process, such as assuming that the image degradation is linear and shift invariant. PIT can be directly adapted to specific degradation models, such as fuzzy kernel, making it more effective in dealing with specific types of degradation. The degradation characteristics of PCB images vary due to differences in manufacturing processes and conditions. PIT is capable of adapting to these changes because it is not based on a fixed transformation basis but rather on the specific information of images and the degradation process itself. Consequently, the application of PIT to enhance the fidelity term of the TV model ensures the preservation of image details and texture information, while simultaneously facilitating the removal of noise during the restoration process. This method demonstrates significant advantages in enhancing IR speed and quality.

6. Dataset details are insufficient

Reply: Thank you for your comments. The data set information has been supplemented. As follows:

The PCB-DD dataset contains 1386 images, covering 6 different types of defects, including pin holes, mouse bites, open circuits, short circuits, dispersion, and miscellaneous copper. These images can be used for image detection, classification, and registration tasks. The images in the dataset are captured by industrial cameras with a resolution of 1920×1080, and the dataset is divided into training, validation, and testing sets in a ratio of 6:2:2. The format of the dataset follows the PASCAL VOC format.

The visual analysis of PCB data set is shown in Fig. 6.

**Figure 6. Analysis chart of pcb number series**

Figures 6(a) and 6(b) show the sample distribution of the PCB dataset and the position of the center of the object. There are 11,601 images in the training set and 1,289 images in the test set. There are 11,601 images in the training set and 1,289 images in the test set.

7. The proposed methodology experimental results are not compared with any state of the art methods.

Reply: Thank you for your advice. The comparison between the proposed method and other methods has been supplemented in the manuscript. The details are as follows:

Finally, the DNN-based PCB-DD method proposed in reference [4] and the CNN-based PCB-DD method proposed in reference [6] are presented and compared with T-YOLOv5. Based on these literatures, the study proposes optimal parameters. The performance is compared and the test is repeated five times to get the t-test results as shown in Table 5.

**Table 5. Performance comparison of different methods**

| Model | Recall | | Testing time | | Accuracy | |
| --- | --- | --- | --- | --- | --- | --- |
|  | *t*-value | *p*-value | *t*-value | *p*-value | *t*-value | *p*-value |
| T-YOLOv5 vs DNN | 2.35 | <0.001 | 3.45 | <0.001 | 2.41 | <0.001 |
| T-YOLOv5 vs CNN | 4.56 | <0.001 | 4.87 | <0.001 | 3.59 | <0.001 |

In Table 5, the *t*-values between the proposed method and other methods are all above 1.96 (at 95% confidence level, two-tailed test). It shows that the differences between different methods are statistically significant. Concurrently, the results illustrate the efficacy and superiority of the proposed method.

6. PLOS authors have the option to publish the peer review history of their article (what does this mean?). If published, this will include your full peer review and any attached files.

If you choose “no”, your identity will remain anonymous but your review may still be made public.

Do you want your identity to be public for this peer review? For information about this choice, including consent withdrawal, please see our Privacy Policy.

Reviewer #1: No

Reviewer #2: No

**We've checked your submission and before we can proceed, we need you to address the following issues:**

1. We note that Figure 7 in your submission contain copyrighted images. All PLOS content is published under the Creative Commons Attribution License (CC BY 4.0), which means that the manuscript, images, and Supporting Information files will be freely available online, and any third party is permitted to access, download, copy, distribute, and use these materials in any way, even commercially, with proper attribution. For more information, see our copyright guidelines: <http://journals.plos.org/plosone/s/licenses-and-copyright.>

We require you to either (1) present written permission from the copyright holder to publish these figures specifically under the CC BY 4.0 license, or (2) remove the figures from your submission:

A. You may seek permission from the original copyright holder of Figure 7 to publish the content specifically under the CC BY 4.0 license.

We recommend that you contact the original copyright holder with the Content Permission Form (<http://journals.plos.org/plosone/s/file?id=7c09/content-permission-form.pdf>) and the following text:
“I request permission for the open-access journal PLOS ONE to publish XXX under the Creative Commons Attribution License (CCAL) CC BY 4.0 (<http://creativecommons.org/licenses/by/4.0/>). Please be aware that this license allows unrestricted use and distribution, even commercially, by third parties. Please reply and provide explicit written permission to publish XXX under a CC BY license and complete the attached form.”

Please upload the completed Content Permission Form or other proof of granted permissions as an ""Other"" file with your submission.

In the figure caption of the copyrighted figure, please include the following text: “Reprinted from [ref] under a CC BY license, with permission from [name of publisher], original copyright [original copyright year].”

B. If you are unable to obtain permission from the original copyright holder to publish these figures under the CC BY 4.0 license or if the copyright holder’s requirements are incompatible with the CC BY 4.0 license, please either i) remove the figure or ii) supply a replacement figure that complies with the CC BY 4.0 license. Please check copyright information on all replacement figures and update the figure caption with source information. If applicable, please specify in the figure caption text when a figure is similar but not identical to the original image and is therefore for illustrative purposes only.

Reply: Thank you for reviewing the manuscript. Figure 7 in the manuscript, which was the subject of a copyright dispute, has been replaced, and the revised images are all compliant with the CC BY 4.0 licence. Corresponding modifications have also been made to the results of the follow-up study. The details are as follows:

Figure 7. Schematic of the natural light verification image used (The above images are from Wikipedia and are similar but not identical to the original images, therefore they are for reference only)

Figure 8. Comparison of PSNR of different methods with different fuzzy kernels

It can be seen from Figure 8(a) that the research proposed algorithm has the highest PSNR value among the four images when the value of FK is taken as 1. Figures 8(b)-(d) also show the reasonableness and effectiveness of the research proposed using the SB algorithm as a PCB image restoration model solving algorithm. The research proposed method has the highest PSNR value in all natural light image processing. The average increase in PSNR obtained by SB algorithm for restoration of 4 images is 0.68% when FK is 1. The average increase in PSNR obtained by restoration of 4 images when FK is 2 is 2.43% compared to TwIST. When FK was 4, SB increased PSNR by an average of 1.35% over TwIST. When FK was 6, SB showed an average increase of 1.46%. Among them, the PSNR value of SB-2 is optimal among the four methods, while the PSNR value of TwIST-2 is also significantly better than TwIST-1. This may be due to the fact that isotropic TV can greatly preserve the details of the image and reduce its distortion when restoring the image, thus obtaining a better image quality for restoring the PCB image. TwIST converges slower, often requires more iterations when dealing with large-scale datasets, and it is more sensitive to noise, which may be the reason why TwIST performs inferior to SB. The recovery times of the four methods with different FKs are shown in Figure 9.

Figure 9. Comparison of time of different methods with different fuzzy kernels

From Figure 9(a)-(d), it can be seen that the time overhead required for the PCB image restoration model proposed in the study is significantly lower than that of TwIST. when FK is 1, the time overhead required for SB is reduced by an average of 39.89% compared to that of TwIST; when FK is 2, the time overhead of SB is reduced by an average of 39.88% compared to that of TwIST; and when FK is 4 and 6, respectively, the time overhead required for SB to in 4 image The time overhead for performing the recovery process is reduced by 38.78% and 40.95% compared to TwIST, respectively. Combining anisotropic TV and isotropic TV also shows that the time overhead of anisotropic TV in performing image restoration is generally lower than that of isotropic TV under the framework of both algorithms, which may be attributed to the fact that the computational complexity required in solving anisotropic TV is lower, and thus the time it requires is reduced accordingly. However, on the whole, the study proposes that the proposed PCB image restoration model is reasonable and effective using the SB algorithm to solve the proposed PCB image restoration model. At the same time, the study further analyses the iterative change of PSMR values when the four methods perform the restoration process by taking image 3 as an example, as shown in Figure 10.
